# Elucidation of puberulic acid–induced nephrotoxicity using stem cell-based kidney organoids

**DOI:** 10.1038/s41598-025-26155-1

**Published:** 2025-11-26

**Authors:** Hiroyuki Nakanoh, Kenji Tsuji, Naruhiko Uchida, Kazuhiko Fukushima, Soichiro Haraguchi, Shinji Kitamura, Jun Wada

**Affiliations:** 1 Department of Nephrology, Rheumatology, Endocrinology and Metabolism, Dentistry and Pharmaceutical Sciences, Okayama University Graduate School of Medicine Dentistry and Pharmaceutical Sciences, Okayama, Japan; 2 Department of Nephrology, Aoe Clinic, Okayama, Japan; 3 Department of Nephrology, Rheumatology, Endocrinology and Metabolism, Okayama University Graduate School of Medicine Dentistry and Pharmaceutical Sciences, 2-5-1 Shikata-cho, Okayama, 700-8558 Japan

**Keywords:** Kidney organoid, Kidney stem cell, Puberulic acid, Nephrotoxicity, Mitochondrial dysfunction, Cell biology, Drug discovery, Medical research, Nephrology, Stem cells

## Abstract

Recent cases of acute kidney injury (AKI) in Japan have been linked to *Beni-koji CholesteHelp* supplements, with puberulic acid identified as a potential nephrotoxic contaminant. To address the need for a reliable in vitro nephrotoxicity testing platform, we developed a screening model using kidney organoids derived from adult rat kidney stem (KS) cells. The organoids were exposed to known nephrotoxicants, including cisplatin and gentamicin, to validate the system. Puberulic acid toxicity was evaluated in both KS cell-derived organoids and wild-type mice. The organoids recapitulated tubular injury induced by known nephrotoxins and showed significant Kim-1 mRNA upregulation. Puberulic acid-treated organoids and mice exhibited morphological features of acute tubular necrosis (ATN), mitochondrial damage, and reduced cytochrome c oxidase subunit IV (COX-IV) expression. Markers of oxidative stress and apoptosis, such as 8-hydroxy-2’-deoxyguanosine (8-OHdG) and cleaved caspase-3, were also elevated. These findings suggest that puberulic acid induces mitochondrial dysfunction and oxidative stress, leading to tubular cell death. Puberulic acid-induced nephrotoxicity was demonstrated using our kidney organoid model. KS cell-derived kidney organoids may provide a simple, reproducible, and rapid platform for nephrotoxicity assessment, which may complement conventional animal experiments.

**Supplementary Information:**

The online version contains supplementary material available at 10.1038/s41598-025-26155-1.

## Introduction

In line with the 3R principles, replacement, reduction, and refinement, organoid-based New Approach Methodologies (NAMs) may serve as alternatives to animal testing in toxicity studies^1^. In the field of nephrotoxicity assessment, kidney organoids derived from induced pluripotent stem (iPS) cells have emerged as a promising in vitro alternative to animal-based models^2,3^. However, their application to the detection of novel or naturally occurring nephrotoxicants remains limited. We have previously established kidney stem (KS) cells derived from a stem/progenitor-like cell line originating from the S3 segment of the renal proximal tubule in adult rats^4^. These KS cells are capable of differentiating into structures resembling both proximal and distal tubule structures in a three-dimensional culture system^5^, partially recapitulating kidney structures.

Recently, acute kidney injury (AKI) associated with *Beni-koji CholesteHelp*, a cholesterol-lowering supplement manufactured by Kobayashi Pharmaceutical and containing red yeast rice (Beni-koji), has emerged as a significant public health concern in Japan^6–10^. Renal biopsies in affected cases revealed tubular injury suggestive of direct nephrotoxicity^10^. A recent investigation by Japan’s the National Institute of Health Sciences and identified that the implicated product batches contained puberulic acid^11^. Further analysis confirmed that this compound can be synthesized by Penicillium adamezioides, a blue mold detected in the production facility^12^. Therefore, Chemical contamination with puberulic acid has been proposed as a potential cause. Puberulic acid was first discovered in cultures of the maize pathogen *Penicillium puberulum*^13^. The chemical formula was C₈H₆O₆ and molecular weight was 198.13 g/mol, structure of puberulic acid was shown in Supplemental Figure S1. Puberulic acid has previously been studied for its antimalarial properties, and earlier reports indicate that it is lethal to mice following two intraperitoneal administrations at 5 mg/kg^14,15^. However, the nephrotoxic properties and underlying mechanisms of puberulic acid remain poorly understood.

In a prior study, we demonstrated that a specific lot of the *Beni-koji CholesteHelp* supplement induced pathological tubular injury in our KS cell-derived kidney organoid model^16^. In the present study, we refined this model to enable quantitative assessment of tubular damage by measuring *Hepatitis A Virus Cellular Receptor 1* (*HAVCR1*), also known as kidney injury molecule-1 (Kim-1), mRNA expression via real-time PCR. Using this system, we investigated the nephrotoxicity of puberulic acid in vitro and further validated the findings in an in vivo mouse model.

## Materials and methods

### Cell culture and organoid formation

The KS cells were isolated from adult male Sprague–Dawley rats as previously described^4^, and were maintained on type I collagen (IWAKI AGC Techno Glass Corporation, Japan) under previously described undifferentiated culture conditions^4^. The procedures for generating KS cell-derived organoids were performed as previously described with minor modifications^5^. Briefly, cells were trypsinized, harvested, and resuspended in medium, ensuring minimal residual trypsin to avoid interference with cell aggregation. Cell suspensions were seeded onto the plates by hanging drop method, with 2.0 × 10⁵ cells in each 25–30 µL droplet per well. The clusters were incubated in 5% CO₂ and 100% humidity at 37 °C for 8 h. For three-dimensional culture, 200 µL of a half-Matrigel solution was applied to Transwell inserts (Corning Life Sciences, USA) and placed into 24-well plates containing 400 µL of differentiation medium. The half-Matrigel solution consisted of a 1:1 mixture of Matrigel and differentiation medium (DMEM/F-12 supplemented with 10% fetal bovine serum (FBS), 250 ng/mL hepatocyte growth factor (HGF), 250 ng/mL glial cell line-derived neurotrophic factor (GDNF), 250 ng/mL basic fibroblast growth factor (bFGF), 250 ng/mL epidermal growth factor (EGF), and 250 ng/mL bone morphogenetic protein-7 (BMP-7). KS cell clusters were transferred into the half-Matrigel solution and cultured at 37 °C with 5% CO₂ and 100% humidity for 2–3 weeks. Nephrotoxic agents (cisplatin, gentamicin and puberulic acid) were applied to the organoids, and were cultured in differentiation medium for 72 h.

### Nephrotoxic agent administration

Puberulic acid (≥ 98% purity, HPLC; serial number NS650101) was purchased from Fujifilm Wako Pure Chemical Corporation (Osaka, Japan). The compound was dissolved in dimethyl sulfoxide (DMSO) and applied to kidney organoids at final concentrations of 2, 10, and 50 µM. Cisplatin (60 µM; Fujifilm Wako Pure Chemical Corporation, Osaka, Japan) and gentamicin (10 mM; Selleck Chemicals, Houston, TX, USA) were used as positive controls.

### Animal experiment

This study was conducted and reported in compliance with the ARRIVE guidelines for animal research reporting. The experimental protocol was approved by the Animal Ethics Review Committee of the Okayama University Graduate School of Medicine, Dentistry and Pharmaceutical Sciences (OKU-2024474). Eight-week-old male C57BL/6 N mice (CLEA Japan, Japan) were housed with free access to tap water and standard laboratory chow. All animals were cared for in accordance with the relevant guidelines and regulations. Mice were randomly assigned to the following two groups (*n* = 6 per group): the control group (CTR) and the puberulic acid group (PUB). Mice in the PUB group were intraperitoneally injected with puberulic acid (5.0 mg/kg body weight) on days 0 and 1. We selected intraperitoneal administration because it is a widely used route in mouse nephrotoxicity models, providing reproducible systemic exposure and technical simplicity compared to subcutaneous injection. Mice in the CTR group were intraperitoneally injected with 100 µL saline on days 0 and 1. On day 4, mice were anesthetized with isoflurane and euthanized by exsanguination via collection of whole blood from the inferior vena cava, in accordance with the approved institutional protocol. The collected blood and kidneys were subsequently harvested for analysis.

### Blood and urine biochemical measurements

Mouse blood samples were collected immediately before euthanizing, and serum creatinine and Blood urea nitrogen (BUN) were measured at the Department of Animal Resources, Advanced Science Research Center, Okayama University. Mouse urine samples were centrifuged to remove cells, cell debris, and other contaminants. Urinary albumin was measured with the Mouse Urinary Albumin Assay Kit (FUJIFILM Wako Pure Chemical Corporation, Japan), and urinary creatinine was measured with a creatinine kit (FUJIFILM Wako Pure Chemical Corporation, Japan).

### Histological and Immunofluorescence

The procedures for histological and immunofluorescence analysis were performed as previously described^17^. Kidney organoids and mouse kidneys were fixed with 10% formalin (Nacalai Tesque, Japan), embedded in paraffin, sectioned at 4 µm, and stained with Hematoxylin and eosin (H&E) or periodic acid-Schiff (PAS). Tubular damage in mouse kidneys was scored by calculation of the percentage of tubules in the corticomedullary junction that displayed tubular dilatation, tubular atrophy, tubular cast formation, and sloughing of tubular epithelial cells or loss of the brush border. The scoring criteria were as follows: 0, none; 1, ≤ 10%; 2, 11–25%; 3, 26–45%; 4, 46–75%; and 5, > 76%. At least 10 high-power fields (magnification, ×200) per section for each sample were examined (n = 6 per group)^18,19^. For immunofluorescence, deparaffinized sections underwent antigen retrieval with EDTA, blocked with 1% BSA-PBS, and incubated with primary antibodies [fluorescein-labeled lotus tetragonolobus lectin (LTL) (Invitrogen), rabbit cleaved caspase-3 (Cell Signaling Technology, USA), and goat Kim-1 (R&D systems, USA) as previously described^20^. For cytochrome c oxidase subunit IV (COX-IV) staining, after antigen retrieval, sections were fixed with 4% paraformaldehyde (PFA) and subsequently permeabilized with 0.1% Triton X-100. For 8-OHdG staining, sections were similarly fixed with 4% PFA following antigen retrieval, then treated with 2 M hydrochloric acid and neutralized with Tris buffer. 4’,6’-diamidino-2-phenylindole (DAPI) (Roche Diagnostic, Switzerland) counterstain were used to visualize nuclei. Imaging was performed with FSX-100 (Olympus, Japan). Cleaved-caspase 3-positive cells per field were evaluated in three kidney organoids tissue sections using five randomly selected images at x200 magnification.

### Transmission electron microscopy (TEM)

TEM was performed as described previously^21^. Briefly, specimens were fixed in 0.1 M cacodylate buffer containing 2.5% glutaraldehyde (pH 7.2) and processed thin sections were stained with uranyl acetate and citrate and examined using a Hitachi H-700 electron microscope.

### Western blotting

Western blotting analysis was performed as previously describe^22^. Briefly, total proteins were extracted using RIPA buffer (Thermo Fisher Scientific, USA) supplemented with protease inhibitors (Promega, USA). Protein concentration was determined using a BCA Protein Assay Kit (Thermo Fisher Scientific, USA). Equal amounts of protein were separated by SDS-PAGE, transferred to nitrocellulose membranes, and blocked with 5% skim milk. Membranes were incubated overnight at 4 °C with primary antibodies [GAPDH (Cell Signaling Technology, USA), Kim-1 (R&D systems, USA)], Cox-IV (Proteintech, USA) and 8-hydroxy-2’-deoxyguanosine (8-OHdG) (Japan Institute for the Control of Aging, Japan), followed by HRP-conjugated secondary antibodies. Signal detection was performed with an enhanced chemiluminescence system (GE Healthcare, USA) and imaged using the Amersham Imager 600. Densitometric analysis was performed using ImageJ software.

### Real-time RT-PCR

RNA isolation and qPCR were performed using the kidney organoids (*n* = 3 per group), as previously described^23^. Total RNA was extracted using an RNA extraction kit (Qiagen Sciences, USA). Real-time PCR was performed on a StepOnePlus™ Real-Time PCR System (Applied Biosystems, USA) using TaqMan Fast Advanced Master Mix (Applied Biosystems, USA). The amplification protocol included an initial enzyme activation at 95 °C for 20 s, followed by 40 cycles of denaturation at 95 °C for 1 s and annealing at 60 °C for 20 s. The comparative threshold cycle (Ct) method was used to calculate fold amplification. Gene expression was analyzed via qPCR using primers specific for HAVCR1 (assay ID: Rn00597703_m, Thermo Fisher Scientific, USA). Appropriate negative and positive controls were included to validate qPCR performance.

### Cell viability analysis

The immortalized human proximal tubular epithelial cell line HK-2 (American Type Culture Collection, USA) was cultured in DMEM/F12 supplemented with 10% FBS, were seeded in 96-well plates and incubated overnight at a density of 5,000 cells per well in 100 µL of medium to adhere. Then, puberulic acid was added at various concentrations (0.4–50 µM) and incubated for 24 h. The cell viability following puberulic acid treatment was assessed using Cell counting kit-8, according to the manufacturer’s protocol (Dojindo Laboratories, Japan), as previously described^24^. Absorbance at 450 nm was measured using a microplate reader (Thermo Fisher Scientific, USA), and cell viability was expressed as a percentage relative to the control group (*n* = 8 per group).

### Statistical analysis

All data are presented as means ± standard error of the mean (SEM). Statistical analysis was performed using JMP software (JMP^®^ 13.2, SAS Institute). Comparisons between two groups were made using either the Student’s t-test or the Wilcoxon rank-sum test, as appropriate. A p-value of < 0.05 was considered as significantly different.

## Results

### Evaluation of tubular injury on kidney organoids

**Fig. 1:**
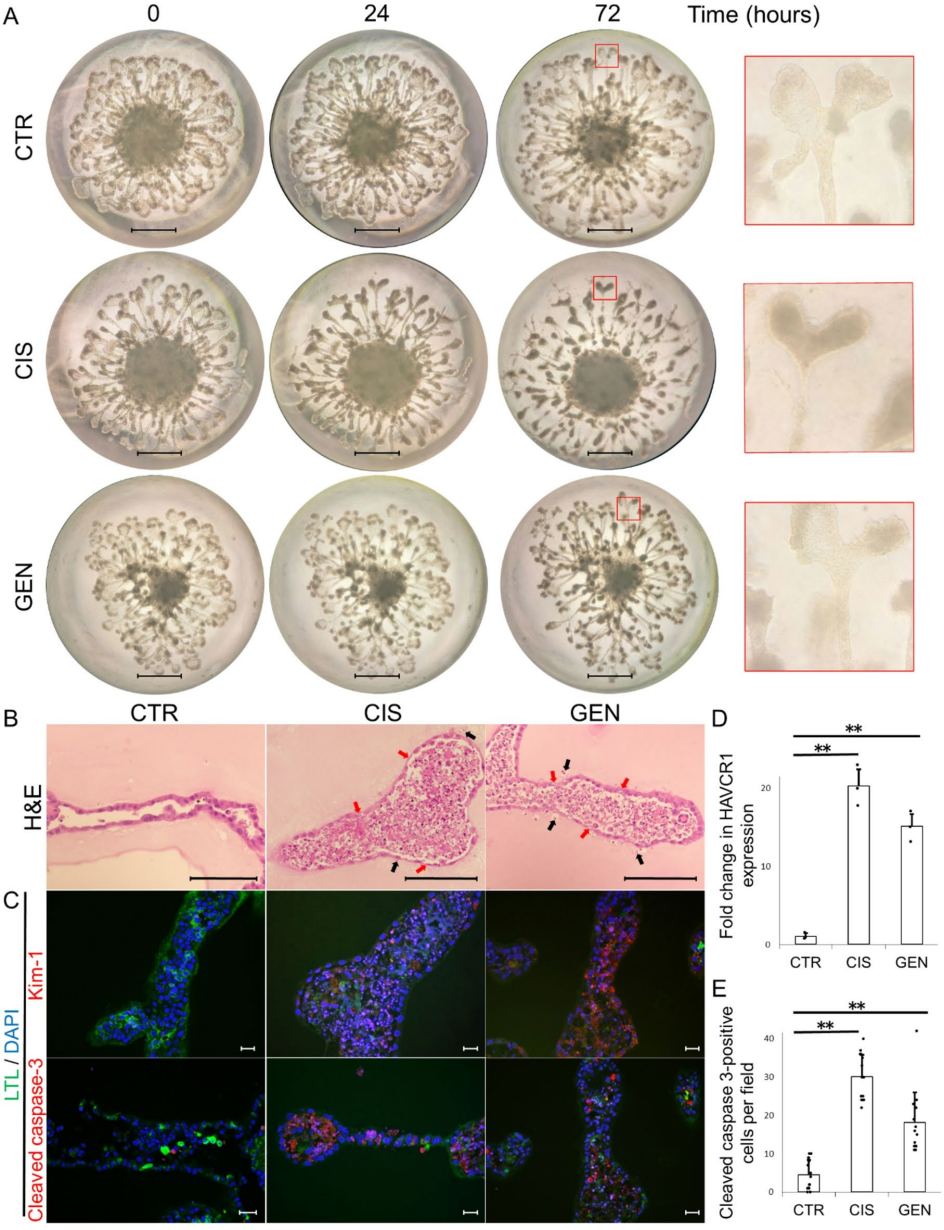
Tubular Injury in Kidney Organoids Induced by Cisplatin and Gentamicin. (**A**) Time-course morphological changes of kidney organoids treated with cisplatin (CIS) or gentamicin (GEN). Organoids were cultured under control conditions (CTR) or exposed to CIS or GEN, and observed at 0, 24, and 72 hours. Morphological alterations such as tubular cell detachment, narrowing, and disruption became progressively evident, particularly in the CIS group at 72 hours. Enlarged images on the far right highlight structural abnormalities. Scale bar, 1 mm. (B-E) Histological and immunofluorescence analysis of kidney organoids after 72-hour treatment. (**B**) Hematoxylin and eosin (H&E) staining of kidney organoids. Tubular epithelial cell detachment (black arrows) and degeneration (red arrows) were observed in both the CIS and GEN groups. (**C**) Immunofluorescence staining for kidney injury molecule-1 (Kim-1, red) and cleaved caspase-3 (red), co-stained with Lotus tetragonolobus lectin (LTL, green) and DAPI (blue). Both Kim-1 and cleaved caspase-3 expression were markedly increased in the CIS and GEN groups compared to CTR. Scale bar, 20 μm. (**D**) Quantitative real-time PCR analysis showing significant upregulation of Kim-1 (*HAVCR1*) mRNA in the CIS and GEN groups. Expression was normalized to Gapdh and presented as fold change relative to CTR (n = 3 per group). (**E**) Quantification of cleaved caspase-3–positive cells per field. Five randomly selected fields from three independent samples were analyzed per group. Data are shown as mean ± SEM. Statistical analysis was performed using Student’s *t*-test. ** P < 0.01.

Kidney organoids were generated using KS cells, and the tubular injury was evaluated following treatment with cisplatin and gentamicin (Fig. 1A). Histological evaluation by stereomicroscopy revealed tubules cell detachment, narrowing and structural disruption in the cisplatin-treated group at 72 h. H&E staining confirmed detachment of tubular epithelial cells, along with cellular and nuclear degeneration in both the cisplatin- and gentamicin-treated groups (Fig. 1B). Immunofluorescence staining demonstrated increased expression of Kim-1 in both treatment groups (Fig. 1C). These findings were further supported by qPCR analysis, which showed significant upregulation of Kim-1 (*HAVCR1*) mRNA expression compared to the control group (Fig. 1D). In addition, immunofluorescence revealed a significant increase in cleaved caspase-3–positive apoptotic cells in the tubules of treated kidney organoids (Fig. 1, C and E).

### Puberulic acid induces kidney organoid damage

**Fig. 2:**
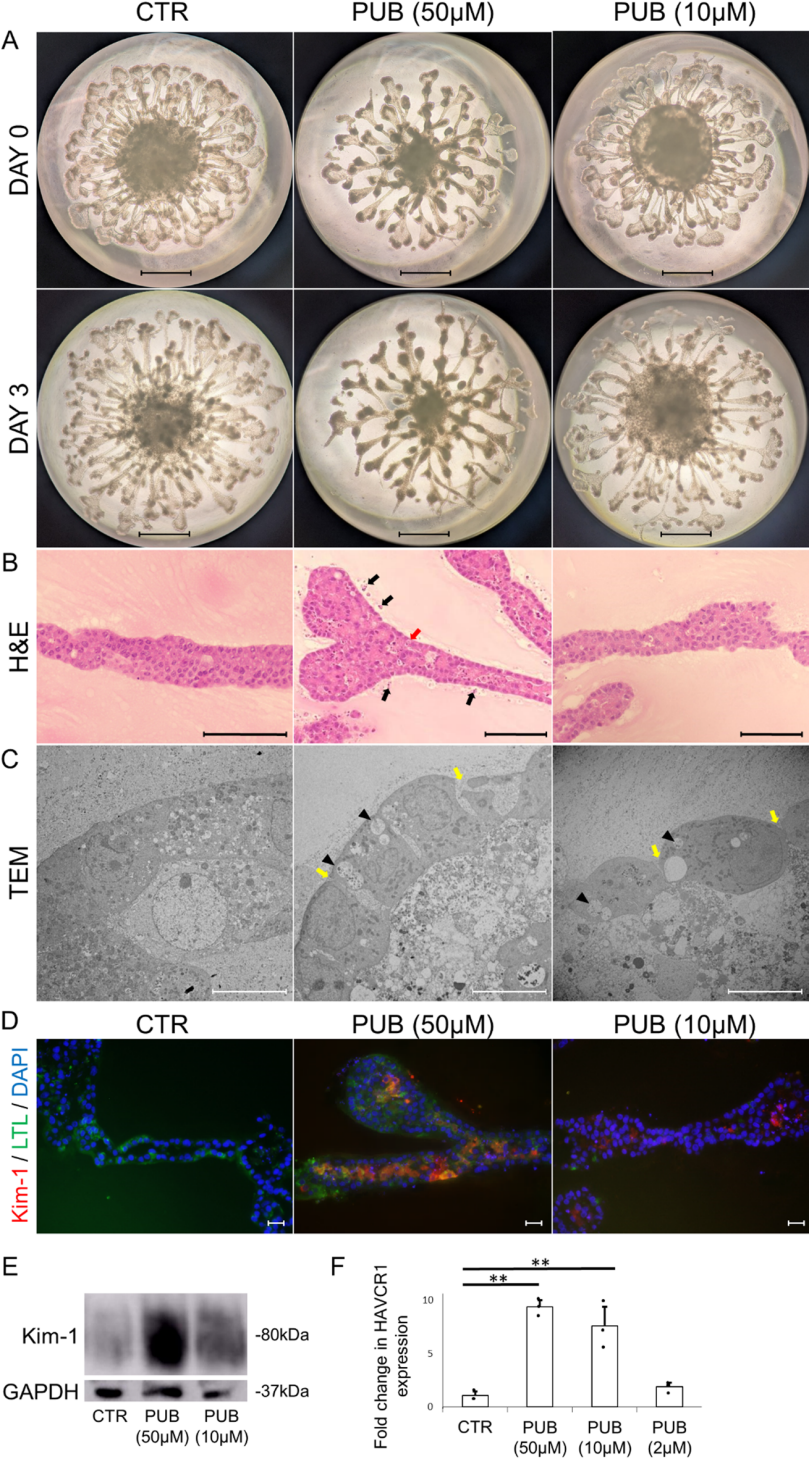
Tubular injury in kidney organoids induced by puberulic acid.

As a first step to evaluate whether the clinically observed nephrotoxicity of puberulic acid is recapitulated in human cells, we assessed cell viability in human proximal tubular epithelial cells (HK-2). HK-2 cells exhibited dose-dependent injury, with significant cytotoxicity observed at a concentration of 50 µM (Supplemental Figure S2). We next utilized the kidney organoid system to further investigate its effects. Treatment with puberulic acid (50 µM) result in tubular detachment, which was evident at 72 h by stereomicroscopy and confirmed by H&E staining (Fig. 2A-B). TEM revealed ultrastructural changes, including loss of intercellular junctions and the presence of low-electron-density vesicles containing remnants of organelles within dilated vacuoles at 10 and 50 µM (Fig. 2C). These morphological changes were consistent with findings previously reported in kidney organoids treated with *Beni-koji Cholestehelp* supplement, and resemble features of drug-induced acute tubular necrosis observed in human kidneys. In addition, Kim-1 expression was elevated in the puberulic acid-treated group, as confirmed by immunofluorescence, western blotting, and qPCR (Fig. 2, D-F).

### Puberulic acid induces acute kidney injury in mice

**Fig. 3:**
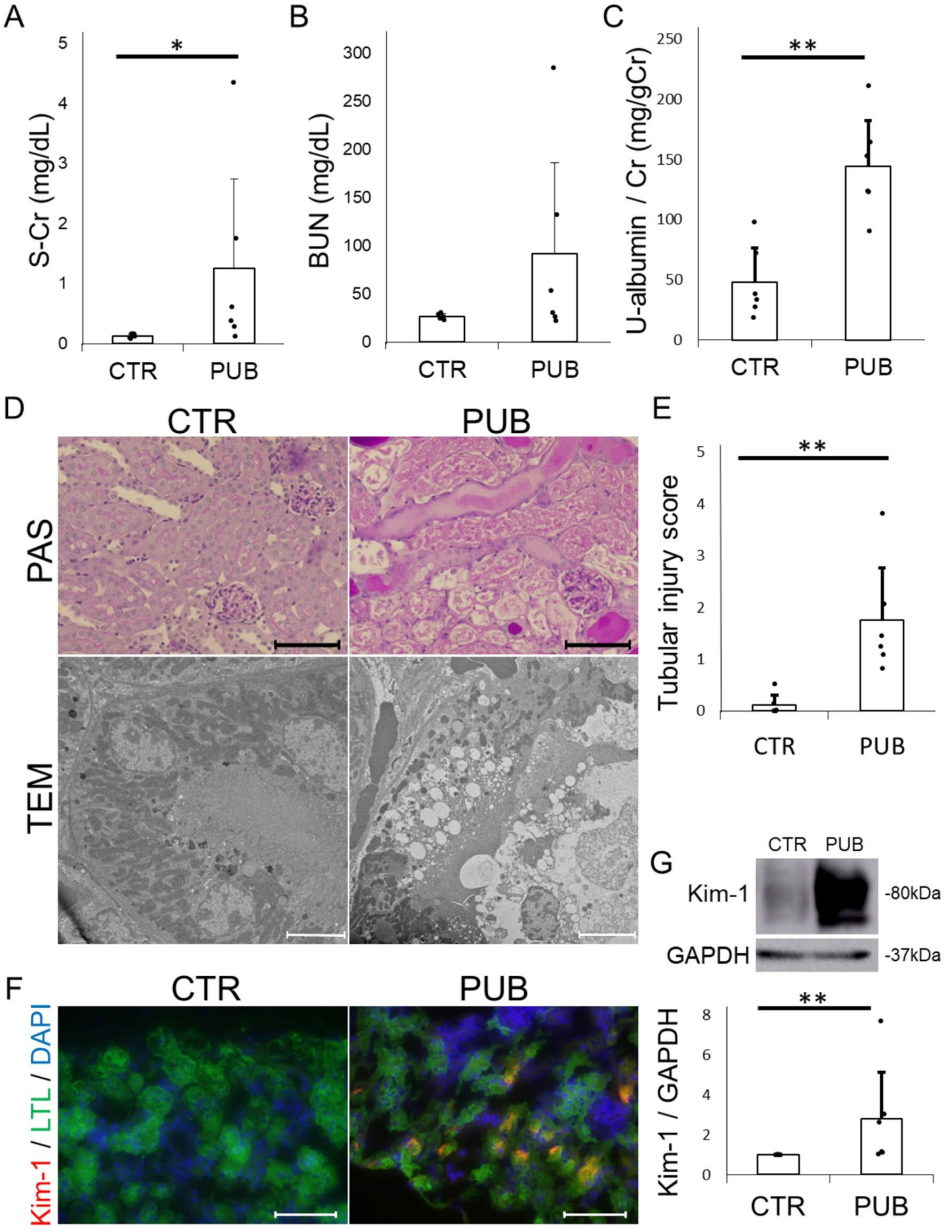
Puberulic acid induces acute kidney injury in mice.

Although our kidney organoids were generated from rat kidneys, we selected mice for in vivo validation for several reasons. First, mice are a widely used species in nephrotoxicity research. Second, previous studies of puberulic acid have been conducted in mice, which facilitates comparison with established dosing paradigms. We administered the compound to C57BL/6 N mice (5.0 mg/kg, on days 0 and 1). Mice treated with puberulic acid (5.0 mg/kg, days 0 and 1) showed elevated levels of serum creatinine and urinary albumin-to-creatinine ratios (Fig. 3A and C). PAS staining revealed tubular atrophy, shedding, and cast formation, suggestive of acute tubular necrosis, while no glomerular abnormalities were evident (Fig. 3D). The renal tubular injury score was significantly higher in the PUB group compared to the CTR group (Fig. 3E). TEM further demonstrated vacuolar degeneration of proximal tubular cells, loss of brush borders, and mitochondrial degeneration in the PUB group, consistent with tubular epithelial cell necrosis (Fig. 3D). In contrast, no ultrastructural abnormalities were detected in the glomeruli, distal tubules, or collecting ducts in either the PUB or CTR groups (Supplemental Figure S3). Moreover, immunofluorescence staining revealed marked upregulation of Kim-1 in the proximal tubules of the PUB group (Fig. 3F), which was further confirmed by western blot analysis (Fig. 3G).

### Puberulic acid induces tubular mitochondrial dysfunction

**Fig. 4:**
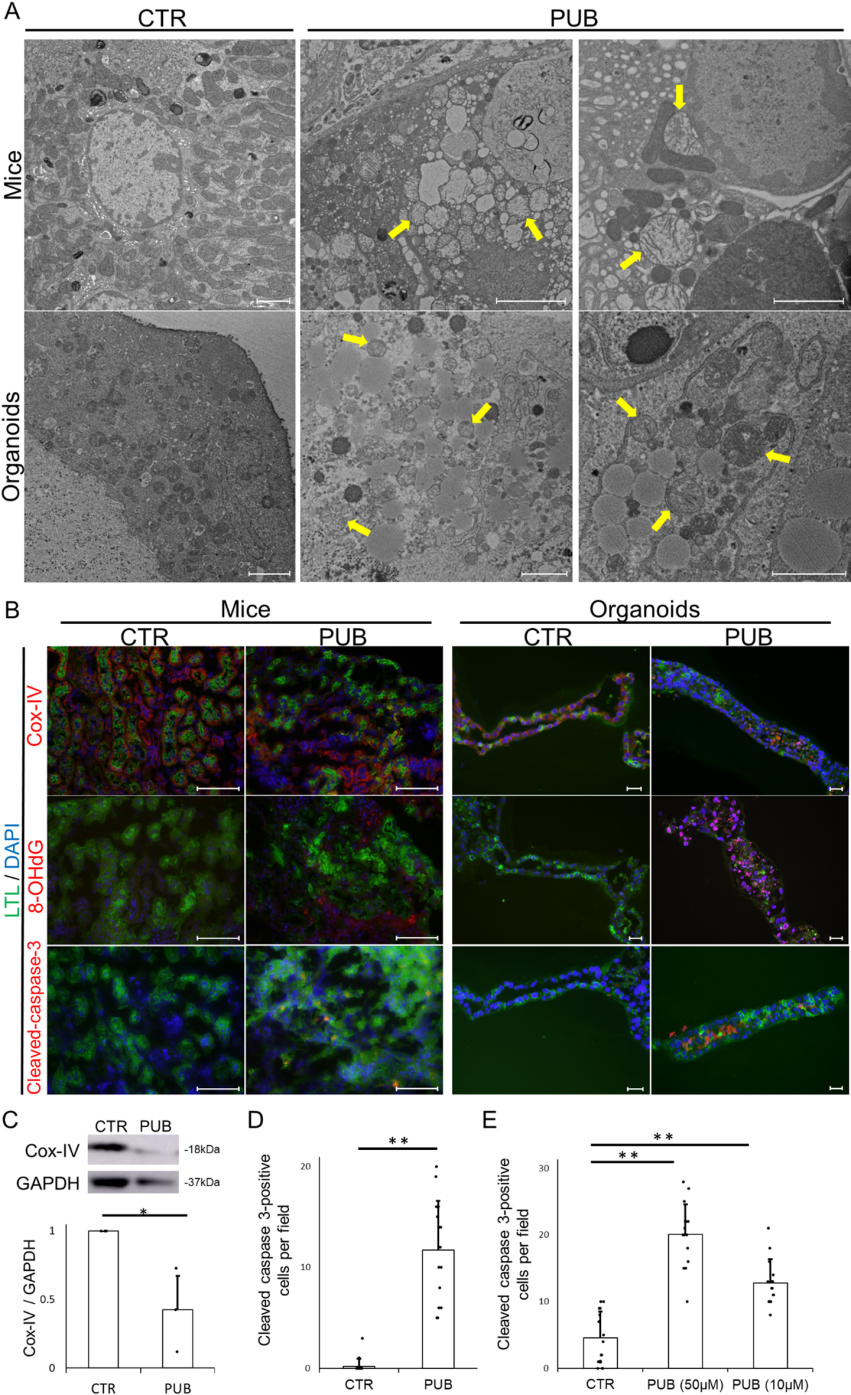
Puberulic Acid Induces Mitochondrial Degeneration. (**A**) Representative transmission electron microscopy (TEM) images of proximal tubular cells from mouse kidneys (upper panels) and kidney organoids (lower panels) in the control (CTR) and puberulic acid–treated (PUB) groups. Organoids treated with puberulic acid (50 µM). In the CTR group, mitochondria were intact with well-defined cristae and organized structure. In contrast, the PUB group showed reduced mitochondrial number and morphological abnormalities such as swelling along with cristae disruption (yellow arrow). Scale bars: 2 μm. (**B**) Representative immunofluorescence images of mouse kidney and kidney organoid stained for cytochrome c oxidase subunit IV (COX-IV, red), 8-hydroxy-2’-deoxyguanosine (8-OHdG, magenta), and cleaved caspase-3 (red) in the CTR and PUB groups. Organoids treated with puberulic acid (50 µM). Lotus tetragonolobus lectin (LTL, green) marks proximal tubules, and nuclei were counterstained with DAPI (blue). Compared to the CTR, the PUB group showed a marked reduction in COX-IV signal intensity, increased accumulation of 8-OHdG, and elevated cleaved caspase-3–positive cells. Scale bars: 100 μm (mice), 20 μm (organoids). (**C**) Western blot analysis of kidney tissue lysates showing decreased COX-IV protein levels in the PUB group compared to the CTR group. GAPDH was used as a loading control. Quantitative densitometry is shown as mean ± SEM (*n* = 3). Statistical analysis was performed using Student’s *t*-test. *P < 0.05. (**D**) Quantification of cleaved caspase-3–positive cells per field in the mice. Data represent mean ± SEM (*n* = 3 mice per group, five fields each). Statistical analysis was performed using Student’s t-test. **P < 0.01. (**E**) Quantification of cleaved caspase-3–positive cells per field in the organoids. Data represent mean ± SEM (*n* = 3 organoids per group, five fields each). Statistical analysis was performed using Student’s t-test. **P < 0.01.

TEM revealed distinct ultrastructural alterations in tubular epithelial cells following puberulic acid treatment. In the CTR group, both mouse kidney and kidney organoid samples exhibited well-preserved mitochondria with intact cristae and an organized cellular architecture. In contrast, samples from the puberulic acid-treated (PUB) group exhibited a notable reduction in mitochondrial number, accompanied by structural abnormalities such as swelling and loss of cristae (Fig. 4A). These findings indicate mitochondrial damage and are consistent with early stage of tubular epithelial cell apoptosis or necrosis. COX-IV immunostaining demonstrated a decrease in mitochondrial signal intensity within the proximal tubules of both mouse kidneys and kidney organoids in the PUB group compared to the CTR group (Fig. 4B). This reduction was further validated by western blot analysis of mouse kidney tissue, which showed significantly decreased COX-IV protein levels in the PUB group (Fig. 4C). To evaluate oxidative stress and apoptosis, immunofluorescence analysis was performed. The PUB group showed increased accumulation of 8-OHdG, a marker of oxidative DNA damage, along with a higher number of cleaved caspase-3–positive cells in both mouse kidney and kidney organoid samples (Fig. 4B, D and E). Collectively, these findings suggest that puberulic acid induces mitochondrial injury, which subsequently triggers oxidative stress and leads to tubular epithelial cell death.

## Discussion

KS cell-derived kidney organoids were previously developed as a screening tool for nephrotoxicity based on morphological assessment. Using this organoid model, we previously reported that cisplatin and specific lots of the *Beni-koji CholesteHelp* supplement induce histopathological tubular injury^16,25^. In those studies, expression of certain transporters, including MATE1 (SLC47A1), was confirmed, although drug transporters such as OCT1/2 (SLC22A1/A2), and OAT1/3 (SLC22A6/A8) were not detected. Therefore, drug uptake in this system remains unclear. Since endocytic function has been demonstrated in iPSC-derived kidney organoids^26^, it is possible that uptake may also occur via endocytic pathways in this model.

We hypothesized that kidney organoids derived from KS cells could serve as a reliable platform to assess nephrotoxicity, particularly for emerging natural toxins such as puberulic acid. In this study, puberulic acid exhibited toxicity in both human proximal tubular epithelial cells and kidney organoids. Furthermore, puberulic acid induced morphological and molecular features consistent with ATN both in vitro and in vivo. These findings suggest that this organoid model is suitable for clinical evaluation of nephrotoxicity. To enhance the reproducibility of nephrotoxicity screening, we introduced qPCR-based quantification of Kim-1 (*HAVCR1*) expression. We demonstrated that our organoid model recapitulated tubular injury induced by known nephrotoxins or puberulic acid, which enabled quantitative evaluation of injury via Kim-1 (*HAVCR1*) expression. This enhancement adds reproducibility and objectivity to the assessment. Previous studies using iPS cell-derived kidney organoids have similarly reported Kim-1 upregulation following gentamicin exposure^27^. Recent advances, including microwell-based automated culture systems, have further expanded the utility of kidney organoids in high-throughput screening for nephrotoxicity and nephroprotection^23^. Compared to iPS cell-derived systems, the KS cell-derived model offers a rapid, reproducible, and cost-effective alternative.

While we demonstrated that specific lots of *Beni-koji CholesteHelp*supplements induced renal tubular injury in this organoid model^16^, its nephrotoxicity remained poorly understood. Similarly, data on the nephrotoxicity and metabolism of puberulic acid remain limited. Recently, it has been suggested that puberulic acid exhibits high affinity for sodium/myo-inositol cotransporter 2 (SLC5A11), implicating a potential toxic mechanism through disruption of osmoregulatory processes in the kidney^28^. In this study, we demonstrated that puberulic acid induces nephrotoxicity, supporting its role as a potential causative agent of AKI associated with specific lot of *Beni-koji CholesteHelp* supplement. Mitochondria generate the energy compound ATP via oxidative phosphorylation, and mitochondrial dysfunction leads to excessive generation of proinflammatory and injurious molecules, such as reactive oxygen species (ROS), inducing oxidative stress that activates both necrotic and apoptotic cell death pathways^29^. This pathogenic cascade has been documented in AKI, including cisplatin-induced nephrotoxicity, which is reported to be associated with a decrease of cytochrome c oxidase (COX) activity and a reduction in COX-IV protein expression, ultimately resulting in respiratory chain dysfunction and increased mitochondrial reactive oxygen species (ROS) production^30^. In this study, mice treated with puberulic acid exhibited acute proximal tubular necrosis and marked mitochondrial structural abnormalities, as demonstrated by PAS staining and TEM. In addition, decreased expression of COX IV and elevated levels of 8-OHdG were observed. These findings suggest that a pathogenic mechanism, similar to that of cisplatin-induced nephrotoxicity, is involved in puberulic acid-induced nephrotoxicity, potentially leading to respiratory chain dysfunction and enhanced mitochondrial ROS production. The kidney organoids closely recapitulated the tubular injury and mitochondrial abnormalities observed in the mouse model, suggesting that they may complement animal experiments in assessing the nephrotoxicity. However, a reliable COX IV band could not be detected in the organoid samples. We infer that limited sample yield and the low abundance of mitochondrial inner-membrane proteins under our extraction conditions contributed to this outcome. Accordingly, we note that limited analyte abundance in organoid samples can preclude reliable Western blot analysis in these assays.

In this study, KS cell-derived kidney organoids and HK-2 cells exhibited different sensitivities to puberulic acid: injury was evident in kidney organoids at 10 µM but not significant in HK-2 cells, whereas both systems demonstrated toxicity at 50 µM. This discrepancy may result from several factors, including transporter immaturity in organoids (lack of OAT1/3 and OCT1/2), species-specific differences between rat-derived organoids and human cells, and structural variation between three-dimensional organoids and two-dimensional cell monolayers, which can influence compound uptake and distribution. For similar reasons, the rationale for using HK-2 cells also has limitations. Although the authors intended to provide a human tubular cells, it should be acknowledged that immortalized HK-2 cells do not fully recapitulate the physiological properties of proximal tubules in vivo. While our results demonstrated dose-dependent toxicity in the organoid model, systematic assessment of time-dependent effects was not performed, and thus the temporal dynamics of puberulic acid-induced nephrotoxicity remain undefined. In vivo, administration of 5.0 mg/kg puberulic acid induced ATN. However, a validated measurement system for puberulic acid has not yet been established; therefore, we did not quantify intrarenal concentrations, and no pharmacokinetic or toxicodynamic data are currently available for this compound. Future studies incorporating renal pharmacokinetic profiling and temporal analysis will be required to determine its distribution, exposure levels, and time- and dose-response, thereby clarifying which experimental platform most closely approximates human nephrotoxicity.

Finally, this kidney organoid system has several limitations. First, these kidney organoids lack the expression of several transporters, including OCT1, OCT2, OAT1, and OAT3^16,25^, consequently drug uptake in this system remains unclear. Accordingly, transporter immaturity may influence toxicity profiling and should be considered an important caveat when interpreting drug specific toxicity. In addition, evidence for drug metabolic capacity in kidney organoids remains very limited. Therefore, systematic characterization of the expression and functional activity of drug metabolizing enzymes in organoids is an important objective for future research. Second, the absence of immune cells, blood vessels, and endothelial components limits the ability to assess kidney damage related to these factors in the kidney, such as tubulointerstitial nephritis, vascular injury, endothelial damage, and ischemia. Third, since toxins are applied externally rather than through physiological routes such as blood circulation and glomerular filtration, direct extrapolation of drug concentrations to clinical settings is not feasible. Incorporating microfluidic systems or perfusable vasculature may help address this gap and improve translational extrapolation of compound concentrations. Despite these limitations, the kidney organoid model remains a potentially valuable in vitro tool for detecting direct tubular toxicity in a controlled environment. It offers a reproducible, scalable, and ethically favorable platform that may complement animal models, and may be particularly useful in the early-phase nephrotoxicity screening. Fourth, our study entails interspecies differences. The organoids were derived from rat cells, the in vivo model used mice, and some cell-based assays employed human cells. Species-specific variation in the expression and activity of drug transporters and drug metabolism enzymes may influence the results. Therefore, the comparisons across systems should be interpreted with considerable caution.

In conclusion, this KS cell-derived kidney organoid model may represent a versatile in vitro platform for nephrotoxicity testing and mechanistic studies. The model successfully recapitulates key morphological and molecular features of tubular injury observed in vivo, including those induced by established nephrotoxins such as cisplatin and gentamicin, as well as the naturally occurring compound puberulic acid. These findings support the potential utility of this model as a tool that may complement animal-based models in preclinical nephrotoxicology. Importantly, the ability to evaluate compound-induced mitochondrial dysfunction, oxidative stress, and cell death pathways in a controlled and scalable system highlights the model’s potential for both hazard identification and mechanistic toxicology. Moreover, this platform aligns with the 3R principles by reducing reliance on animal testing, thereby addressing both ethical and regulatory demands for more human-relevant toxicity assessment systems. Future research should focus on enhancing the physiological relevance of the model, for example by incorporating vascular, immune, and transporter components. In addition, integration with high-throughput and microfluidic technologies may further broaden its utility in drug development and toxicology. With further development, this model may serve as a valuable tool for both predicting human nephrotoxicity and informing personalized and regulatory approaches in toxicology.

## Supplementary Information

Below is the link to the electronic supplementary material.

Supplementary Material 1

## Data Availability

All data generated or analyzed during this study are included in this published article and its supplementary information files.

## References

[1] Poh, W. T. & Stanslas, J. The new paradigm in animal testing - 3Rs alternatives. *Regul. Toxicol. Pharmacol.* **153**, 105705. 10.1016/j.yrtph.2024.105705 (2024). 39299677 10.1016/j.yrtph.2024.105705

[2] Czerniecki, S. M. et al. High-Throughput Screening Enhances Kidney Organoid Differentiation from Human Pluripotent Stem Cells and Enables Automated Multidimensional Phenotyping. *Cell Stem Cell* **22**, 929–940. 10.1016/j.stem.2018.04.022 (2018). 29779890 10.1016/j.stem.2018.04.022 PMC5984728

[3] Oishi, H., Tabibzadeh, N. & Morizane, R. Advancing preclinical drug evaluation through automated 3D imaging for high-throughput screening with kidney organoids. *Biofabrication*10.1088/1758-5090/ad38df (2024). 38547531 10.1088/1758-5090/ad38df PMC11304660

[4] Kitamura, S. et al. Establishment and characterization of renal progenitor like cells from S3 segment of nephron in rat adult kidney. *FASEB J.* **19**, 1789–1797. 10.1096/fj.05-3942com (2005). 16260649 10.1096/fj.05-3942com

[5] Kitamura, S., Sakurai, H. & Makino, H. Single adult kidney stem/progenitor cells reconstitute three-dimensional nephron structures in vitro. *Stem Cells*. **33**, 774–784. 10.1002/stem.1891 (2015). 25422083 10.1002/stem.1891

[6] Uchiyama, K. et al. Acute kidney injury associated with red yeast rice (Beni-koji) supplement: A report of two cases. *Kidney Med.* **6**, 100908. 10.1016/j.xkme.2024.100908 (2024). 39507393 10.1016/j.xkme.2024.100908 PMC11539353

[7] Chikasue, A. et al. Three cases of red yeast Rice-Containing Supplement-Induced acute kidney injury and Fanconi syndrome. *Am. J. Kidney Dis.* **85**, 522–526. 10.1053/j.ajkd.2024.08.007 (2025). 39424254 10.1053/j.ajkd.2024.08.007

[8] Habuka, M. et al. Fanconi syndrome with acute proximal tubular injury induced by a dietary supplement containing beni-koji: a case series report. *BMC Nephrol.* **25**, 446. 10.1186/s12882-024-03903-5 (2024). 39639196 10.1186/s12882-024-03903-5 PMC11622456

[9] Abe, M., Ogawa, T., Magome, N., Ono, Y. & Tojo, A. Element analysis applied to investigate acute kidney injury induced by red yeast rice supplement. *Med. Mol. Morphol.* **58**, 53–61. 10.1007/s00795-024-00411-1 (2025). 39535557 10.1007/s00795-024-00411-1 PMC11829840

[10] Shinzawa, M. et al. A nationwide questionnaire study evaluated kidney injury associated with Beni-koji tablets in Japan. *Kidney Int.* **107**, 530–540. 10.1016/j.kint.2024.11.027 (2025). 39708997 10.1016/j.kint.2024.11.027

[11] Tanaka, S. et al. Novel compounds isolated from health food products containing beni-koji (red yeast rice) with adverse event reports. *J. Nat. Med.* **78**, 845–848. 10.1007/s11418-024-01827-w (2024). 38834898 10.1007/s11418-024-01827-w

[12] Yoshinari, T. et al. Mechanism of puberulic acid contamination in red yeast rice tablets that caused a serious food poisoning outbreak in Japan. *Proc. Jpn Acad. Ser. B Phys. Biol. Sci.*10.2183/pjab.101.017 (2025). 40159186 10.2183/pjab.101.017 PMC12332415

[13] Birkinshaw, J. H. & Raistrick, H. Studies in the biochemistry of micro-organisms: puberulic acid C(8)H(6)O(6) and an acid C(8)H(4)O(6), new products of the metabolism of glucose by penicillium puberulum Bainier and penicillium aurantio-virens Biourge. With an appendix on certain dihydroxybenzenedicarboxylic acids. *Biochem. J.* **26**, 441–453. 10.1042/bj0260441 (1932). 16744843 10.1042/bj0260441 PMC1260924

[14] Iwatsuki, M. et al. In vitro and in vivo antimalarial activity of puberulic acid and its new analogs, viticolins A-C, produced by penicillium sp. FKI-4410. *J. Antibiot. (Tokyo)*. **64**, 183–188. 10.1038/ja.2010.124 (2011). 21063422 10.1038/ja.2010.124

[15] Sennari, G. et al. Antimalarial troponoids, puberulic acid and viticolins; divergent synthesis and structure-activity relationship studies. *Sci. Rep.* **7**, 7259. 10.1038/s41598-017-07718-3 (2017). 28775291 10.1038/s41598-017-07718-3 PMC5543150

[16] Nakanoh, H. et al. Supplement-induced acute kidney injury reproduced in kidney organoids. *Am. J. Nephrol.*10.1159/000544795 (2025). 39978331 10.1159/000544795 PMC12342697

[17] Sang, Y. et al. Semaporin3A inhibitor ameliorates renal fibrosis through the regulation of JNK signaling. *Am. J. Physiol. Ren. Physiol.* **321**, F740–F756. 10.1152/ajprenal.00234.2021 (2021). 10.1152/ajprenal.00234.2021 34747196

[18] Tsuji, K., Kitamura, S., Sang, Y., Fukushima, K. & Wada, J. Adult kidney stem/progenitor cells contribute to regeneration through the secretion of trophic factors. *Stem Cell. Res.* **46**, 101865. 10.1016/j.scr.2020.101865 (2020). 32505897 10.1016/j.scr.2020.101865

[19] Dong, Y. et al. Ischemic duration and frequency determines AKI-to-CKD progression monitored by dynamic changes of tubular biomarkers in IRI mice. *Front. Physiol.* **10**, 153. 10.3389/fphys.2019.00153 (2019). 30873045 10.3389/fphys.2019.00153 PMC6401609

[20] Pileri, S. A. et al. Antigen retrieval techniques in immunohistochemistry: comparison of different methods. *J. Pathol.* **183**, 116–123 (1997). 9370957 10.1002/(SICI)1096-9896(199709)183:1<116::AID-PATH1087>3.0.CO;2-2

[21] Fukushima, K., Kitamura, S., Tsuji, K., Sang, Y. & Wada, J. Sodium glucose co-transporter 2 inhibitor ameliorates autophagic flux impairment on renal proximal tubular cells in obesity mice. *Int. J. Mol. Sci.*10.3390/ijms21114054 (2020). 32517059 10.3390/ijms21114054 PMC7312919

[22] Sang, Y. et al. Semaphorin3A-inhibitor ameliorates doxorubicin-induced podocyte injury. *Int. J. Mol. Sci.*10.3390/ijms21114099 (2020). 32521824 10.3390/ijms21114099 PMC7312798

[23] Tsuji, K., Kitamura, S. & Makino, H. Hypoxia-inducible factor 1alpha regulates branching morphogenesis during kidney development. *Biochem. Biophys. Res. Commun.* **447**, 108–114. 10.1016/j.bbrc.2014.03.111 (2014). 24690177 10.1016/j.bbrc.2014.03.111

[24] Xing, D., Ma, Y., Lu, M., Liu, W. & Zhou, H. Paeoniflorin alleviates hypoxia/reoxygenation injury in HK-2 cells by inhibiting apoptosis and repressing oxidative damage via Keap1/Nrf2/HO-1 pathway. *BMC Nephrol.* **24**, 314. 10.1186/s12882-023-03366-0 (2023). 37884904 10.1186/s12882-023-03366-0 PMC10601317

[25] Ueno, S. et al. Kidney organoid derived from renal tissue stem cells is a useful tool for histopathological assessment of nephrotoxicity in a cisplatin-induced acute renal tubular injury model. *J. Toxicol. Pathol.* **35**, 333–343. 10.1293/tox.2022-0006 (2022). 36406172 10.1293/tox.2022-0006 PMC9647211

[26] Sahara, Y., Fukui, C., Kuniyoshi, Y. & Takasato, M. Proximal tubule cell maturation rate and function are controlled by PPARalpha signaling in kidney organoids. *Commun. Biol.* **7**, 1532. 10.1038/s42003-024-07069-6 (2024). 39604738 10.1038/s42003-024-07069-6 PMC11603349

[27] Morizane, R. et al. Nephron organoids derived from human pluripotent stem cells model kidney development and injury. *Nat. Biotechnol.* **33**, 1193–1200. 10.1038/nbt.3392 (2015). 26458176 10.1038/nbt.3392 PMC4747858

[28] Hayama, T., Sugawara, R., Kamata, R., Sekijima, M. & Takeda, K. Comprehensive molecular Docking on the AlphaFold-predicted protein structure proteome: identifying target protein candidates for puberulic acid. *J. Toxicol. Sci.* **50**, 309–324. 10.2131/jts.50.309 (2025). 40603041 10.2131/jts.50.309

[29] Emma, F., Montini, G., Parikh, S. M. & Salviati, L. Mitochondrial dysfunction in inherited renal disease and acute kidney injury. *Nat. Rev. Nephrol.* **12**, 267–280. 10.1038/nrneph.2015.214 (2016). 26804019 10.1038/nrneph.2015.214 PMC5469549

[30] Zsengeller, Z. K. et al. Cisplatin nephrotoxicity involves mitochondrial injury with impaired tubular mitochondrial enzyme activity. *J. Histochem. Cytochem.* **60**, 521–529. 10.1369/0022155412446227 (2012). 22511597 10.1369/0022155412446227 PMC3460350

