# Supplementary material for: Elucidation of puberulic acid–induced nephrotoxicity using stem cell-based kidney organoids
Source: Sci Rep. 2025 Nov 26;15:42195. doi: 10.1038/s41598-025-26155-1 (PMC12658070; doi:10.1038/s41598-025-26155-1)
Supplement: Supplementary file 1 — Supplementary Material 1 [file 41598_2025_26155_MOESM1_ESM.pdf]

Supplementary Material for:
Elucidation of Puberulic Acid–Induced Nephrotoxicity Using Stem Cell-based Kidney Organoids

Table of contents

Figure S1. Chemical Structure of Puberulic Acid..2

Figure S2. Cytotoxicity of Puberulic Acid on Proximal Tubular Epithelial Cells...............3

Figure S3. Ultrastructural analysis of renal structures………………………........…………....4

Figure S4. Full-length blots in Figure 2E…………………………………........…………....5

Figure S5. Full-length blots in Figure 3G…………………………………........………....6

Figure S6. Full-length blots in Figure 4C…………………………………........……......7

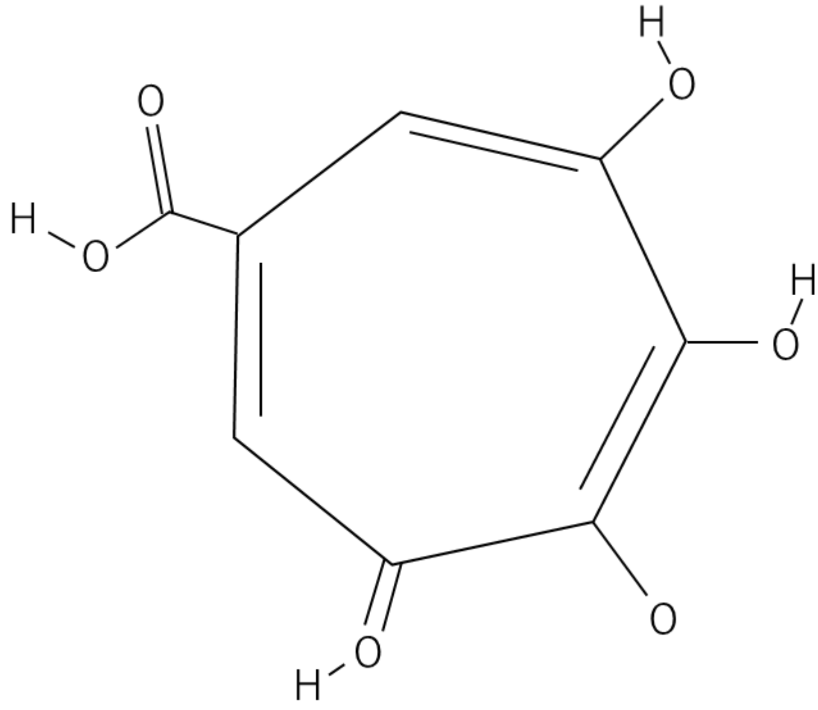

Figure S1. Chemical Structure of Puberulic Acid

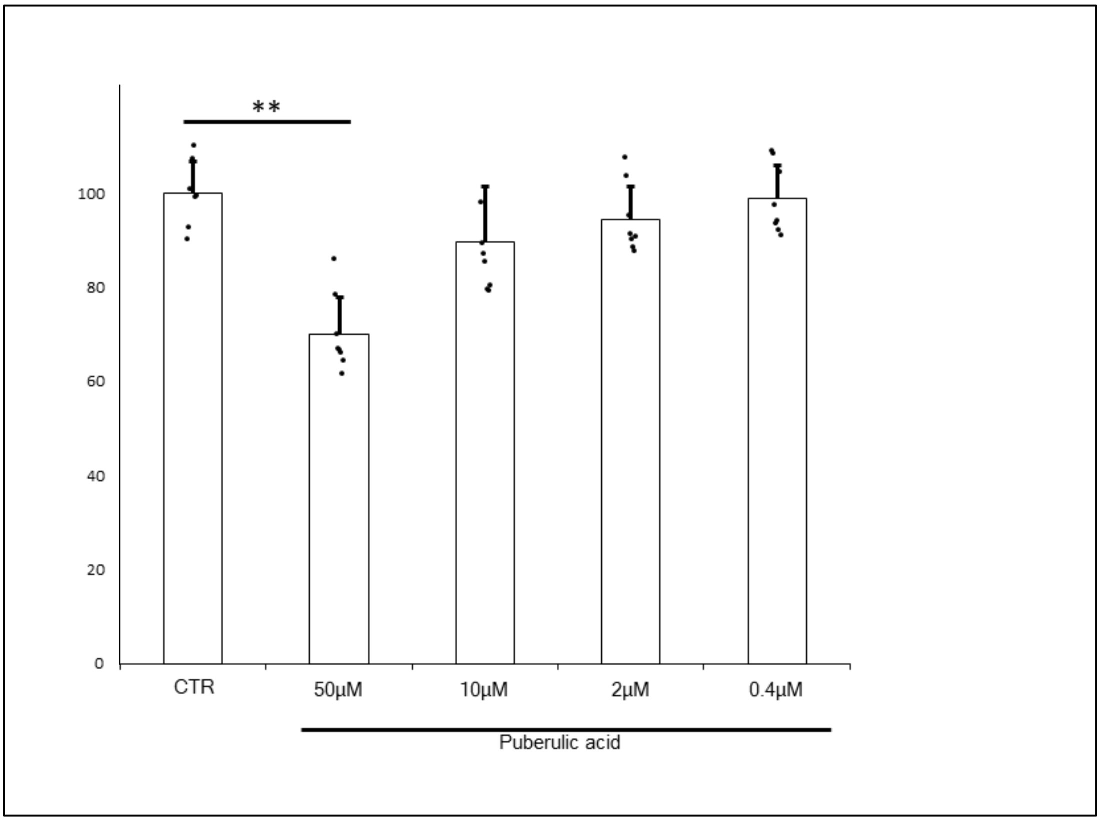

Figure S2. Cytotoxicity of Puberulic Acid on Proximal Tubular Epithelial Cells

Cytotoxicity assay was conducted in HK-2 cells treated with puberulic acid at concentrations of 0.4, 2, 10, and 50 µM. Cell viability was significantly reduced at 50 µM compared to the control group (CTR) (n = 7–8 per group). Data are presented as mean ± SD. Statistical analysis was performed using Student's t-test. ** $p<0.01$.

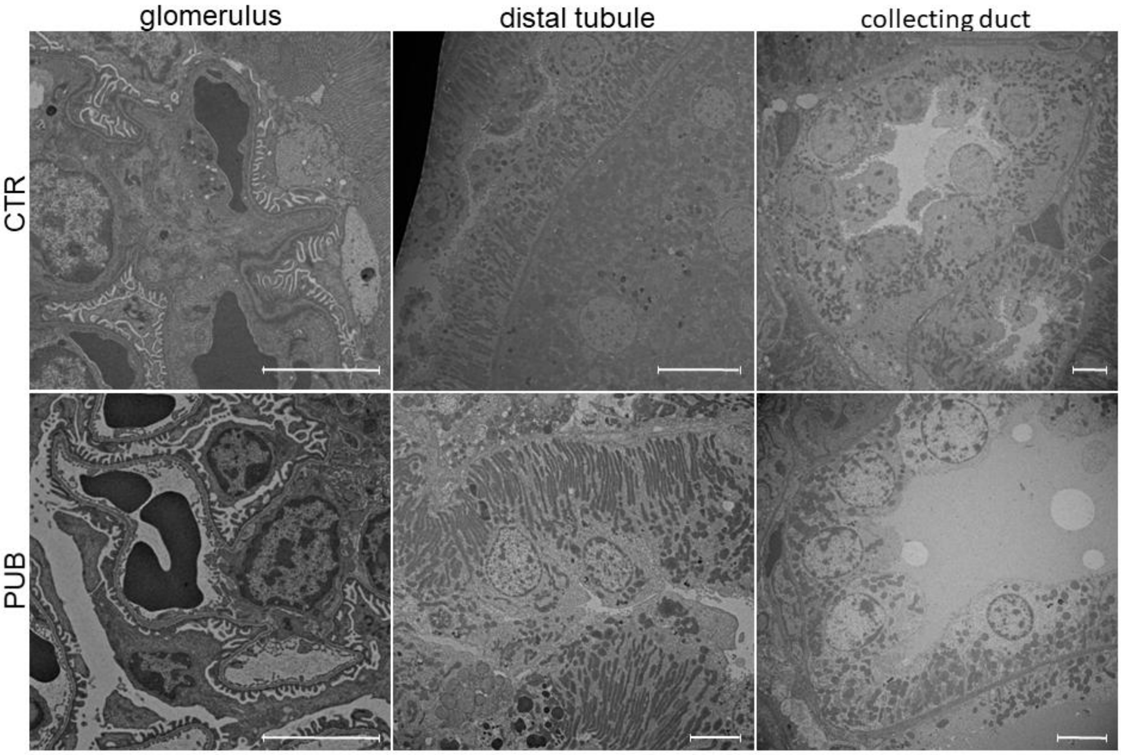

Figure S3. Ultrastructural analysis of glomerulus, distal tubule, and collecting duct

Representative images of transmission electron microscopy images of the glomerulus, distal tubule, and collecting duct in mouse kidney under control conditions (CTR) and after puberulic acid (PUB) treatment, showing no ultrastructural abnormalities in these regions. Scale bars: 5 μm.

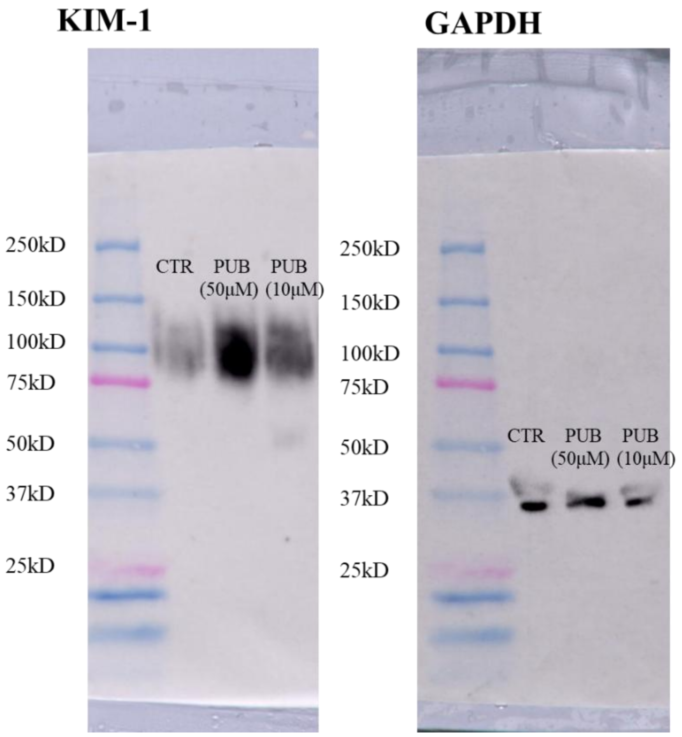

Figure S4. Full-length blots in Figure 2E

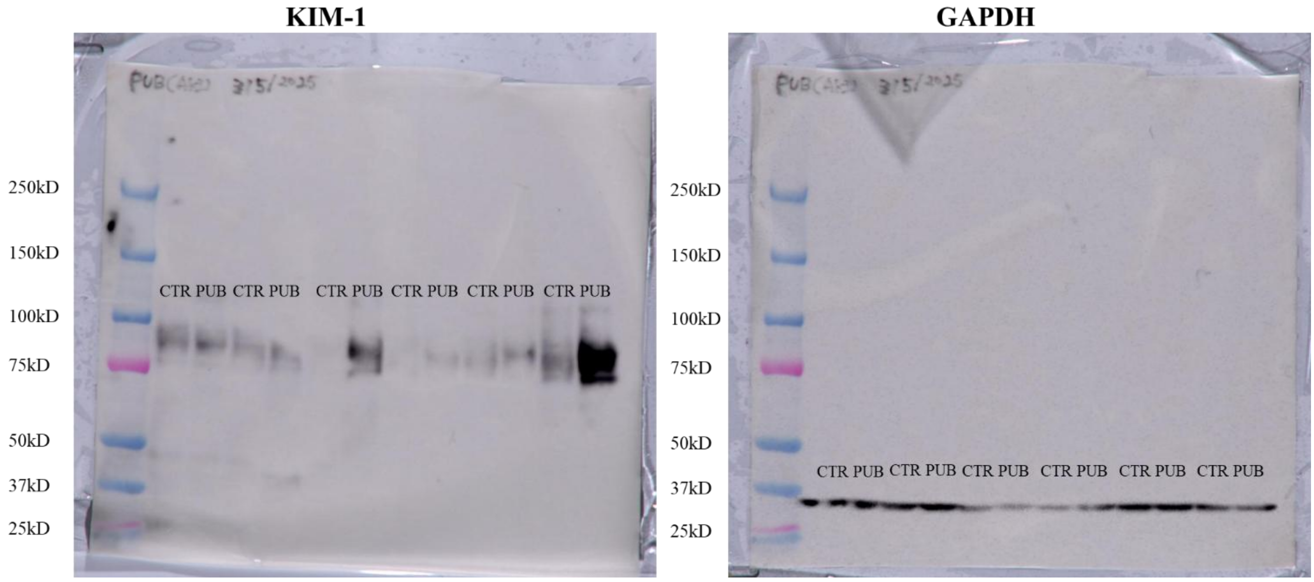

Figure S5. Full-length blots in Figure 3G

COX-IV

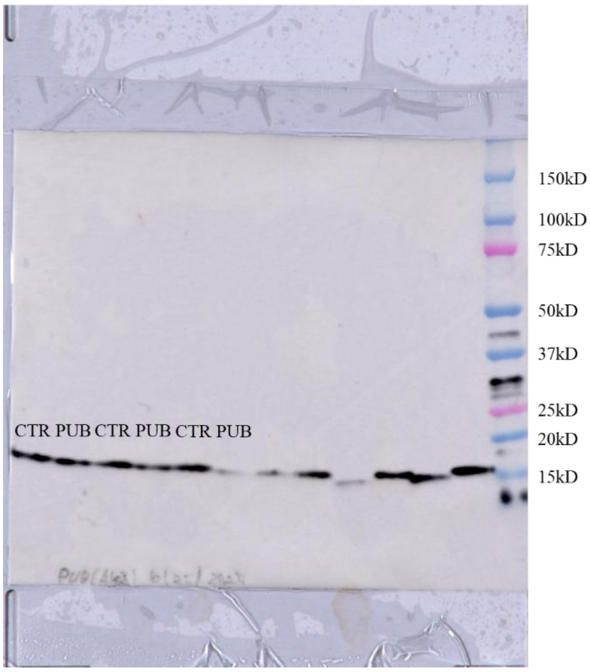

GAPDH

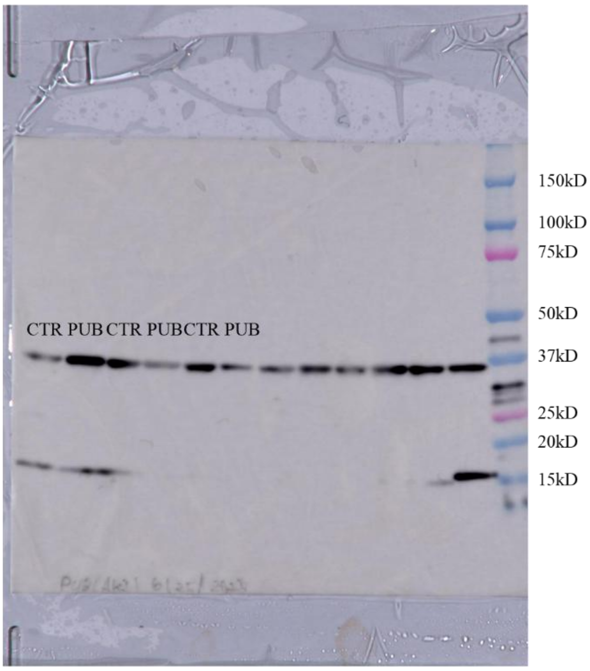

Figure S6. Full-length blots in Figure 4C
